# “I feel like marijuana is the only drug that wouldn’t kill me”: perceptions of cannabis use in previously incarcerated Black men who have sex with other men

**DOI:** 10.1186/s12954-023-00744-7

**Published:** 2023-02-03

**Authors:** Rey Flores, Jared Kerman, John Schneider, Nina Harawa

**Affiliations:** 1 University of Chicago, 5801 S Ellis Ave, Chicago, IL 60637 USA; 2 Chicago Center for HIV Elimination, 1525 E. 55th St. Suite 205, Chicago, IL 60637 USA; 3 University of Illinois at Chicago, 750 S Halsted St., Chicago, IL 60607 USA; 4 David Geffen School of Medicine at UCLA, 1100 Glendon Ave. Suite 850, Los Angeles, CA 90024 USA; 5 Charles Drew University of Medicine and Science, 1731 E. 120th St., Los Angeles, CA 90059 USA

**Keywords:** Marijuana, Cannabis, Black MSM, Incarceration, Substance use, Social network

## Abstract

**Background:**

Fragmented state laws have impacted cannabis uptake and perceptions in the USA. Little research has explored the attitudes, beliefs, and social network influences of young Black men who have sex with men (BMSM) who have experienced incarceration and use cannabis. While problematic cannabis use is not well defined and understudied, scholars have found that a person’s social network can mediate problematic substance use and reduce recidivism rates by providing both tangible and emotional support. This analysis examines how social networks contribute to cannabis perceptions and use among BMSM with criminal legal system involvement in Chicago, IL, and Houston TX.

**Methods:**

Researchers conducted interviews with 25 cis gender Black men informed by life course theory, with a focus on the role of social networks, incarceration, and other life experiences in substance use. All interviews were audio-recorded, de-identified, and transcribed; participants were compensated $50. A deductive-inductive thematic analysis was used to analyze all qualitative data collected.

**Results:**

Twelve BMSM in Chicago and 13 BMSM in Houston (*M* = 26.6 years old, SD = 3.7) were interviewed. A majority identified as gay (56%), with 12 participants (48%) reporting having a high school diploma or equivalent; their average age of first substance use was 15.2 (SD = 2.9). Participants perceived cannabis usage to be categorically distinct from other intoxicating substance usage, with many describing it as not harmful and potentially beneficial. Three themes shaped their choices and attitudes regarding cannabis and “hard” drugs—*social networks*, *need fulfillment*, and *knowledge of risk*.

**Conclusion:**

Participant descriptions of cannabis use emphasize their drug-use behavior as being produced by agent decision-making and risk assessment. Future work should expand on how these decisions are made, and how social networks can be leveraged to encourage non-harmful drug consumption behaviors.

## Background

Over the past decade, perceptions of cannabis and cannabis use have changed radically, with 37 states and D.C. legalizing medical usage and 21 states and D.C. allowing adult recreational usage as of January of 2023 [1]. In 2019, Illinois became the 11th state to legalize recreational cannabis for adult use, and the first in the country to adopt a regulatory system for cannabis cultivation, testing, and sales [2]. Texas is one of 13 states without a comprehensive medical cannabis law, only allowing patients with specific debilitating medical conditions to access low-THC (< 1%) medical cannabis products [3]. In May of 2021 the U.S. House of Representative introduced the “Marijuana Opportunity Reinvestment and Expungement Act” that would legalize cannabis and expunge federal cannabis arrest and offenses from individuals' records [4]. This bill has huge implications for Black communities who are disproportionately impacted by incarceration for cannabis-related offenses [5, 6]. Recent reports estimate Black people are 3.64 times as likely as their white peers to be arrested for cannabis possession despite similar rates of cannabis consumption [6, 7].

Research has explored the risks associated with cannabis usage in Black men who have sex with men (BMSM) such as homelessness, incarceration, and high risk sexual behaviors [8, 9]. Cannabis and sexual risk behaviors maintain a complex relationship as cannabis is often co-used with alcohol and other illicit substances [8]. In one study people who used cannabis heavily were more likely to be unaware of their human immunodeficiency virus (HIV) status; whereas, associations with other HIV outcomes were inconclusive [9]. Sociostructural factors that contribute to substance use-related risk factors, such as incarceration [10, 11], community context (e.g., prevalence of drug use, poverty) [12, 13], and social networks [14, 15], are gaining increasing research focus. Social networks contribute to the context and uptake of drug use via modeling and the sharing of information on how to use substances, drug procurement, and how to sustain usage [16–20]. Previous studies show that Black men report learning about cannabis from peers and family members [16] and use cannabis to cope with racial discrimination [16, 21, 22], financial challenges [23], pain, and other stressors [24, 25]; but the perceptions and attitudes toward cannabis use and other “harder” drugs are not well described.

Whether consumed for self-medicative or recreational purposes, many participants in prior studies endorse daily usage as being a non-problematic, normalized, and a beneficial part of their lives [12, 16, 26]. Many providers utilize the Diagnostic and Statistical Manual of Mental Disorders fifth edition (DSM-5) to determine problematic cannabis use, and assessments are supported by varied metrics and assessment tools [27, 28]. The DSM-5 determines a person may be diagnosed with cannabis use disorder if they meet two of their designated nine criteria that primarily evaluate ability to cease/decrease cannabis use, frequency and amount consumed, and impairment of ability to achieve daily tasks [29]. A person’s social network can influence that person to use substances in ways that are problematic, but these networks have also been found to mediate problematic substance use and recidivism [10, 30, 31] by supporting persistent recovery [11, 15, 32].

By characterizing social networks and contexts to inform the development of effective interventions, researchers and policymakers can leverage social structures for behavioral change [33–36]. To generate further understanding of this topic as well as support potential interventions, this paper explores how one’s social network contributes to cannabis perception and usage patterns among BMSM who have been involved with the criminal legal system.

## Methods

These data were collected via a supplement to a NIDA-funded three-site study designed to develop agent-based models to examine the impact of various interventions on HIV in BMSM who experience incarceration. The parent study involved three of the four largest jail systems in the country (Chicago, Houston, and Los Angeles). Interviews from Chicago and Houston were included in this analysis because they were collected first using similar interviewing approaches. Los Angeles data collection was incomplete at the time of this paper and used more in-depth approaches, building on what was learned from the Chicago and Houston interviews.

### Dataset and sample

The study utilizes life course interviews [37, 38] with BMSM who have been incarcerated at least once in their lifetime. We used these data to qualitatively examine perceptions of cannabis use in BMSM in Chicago, IL, and Houston, TX. Eligibility criteria were: (1) age 18–34 years old; (2) Black or African American identified; (3) cis-gender male; (4) had sex with a man in the 12 months prior to their interview; (5) incarcerated for at least 24 h; and (6) misuse of opioids or use of illegal drugs other than opioids or marijuana in their lifetime. Researchers conducted 25 interviews that lasted approximately 60 min each using a semi-structured interview guide to explore participants’ experiences with substances, social networks, and incarceration. Participants received a $50 incentive. University of Chicago institutional review board provided oversight to all study procedures.

### Analytical approach

Interviews were audio-recorded and transcribed, then coded and analyzed. Minority Stress Theory [39, 40] and the Social Ecological Theory [41] were used to understand how contextual factors (i.e., familial substance use) produce a social environment that encourages sustained or exacerbated substance misuse. By using a modified version of Braun and Clarke’s six-phase guide to a deductive-inductive thematic analysis [42], we systematically identify and offer insights into themes observed across the sample. Dedoose (Sociocultural Research Consultants, 2018) was used for data coding and management.

## Results

Twelve BMSM in Chicago and 13 BMSM in Houston (*n* = 25) were interviewed. A majority identified as gay (56%), with 12 participants (48%) reporting having a high school diploma or equivalent; their average age of first substance use was 15.2 years (SD = 2.9) (see Table 1). Participants perceived cannabis to be categorically distinct from other intoxicating substances. Participant attitudes toward cannabis compared to “hard” or “heavy” substances, such as methamphetamine, cocaine, ecstasy, and opiates (i.e., heroine and prescription pills), were shaped by a wide range of factors, including their social network, need fulfillment, and knowledge of risk.

**Table 1:** Demographics and substance use among BMSM in Chicago IL, and Houston TX, *n* = 25; year 2019–2021

	*N* (%)
Age (M, SD)	26.6 (3.7)
Site location	
Chicago	12 (48%)
Houston	13 (52%)
Sexual orientation	
Gay or homosexual	14 (56%)
Bisexual	7 (28%)
Straight or heterosexual	4 (16%)
Education	
K-12	6 (24%)
GED	2 (8%)
High school diploma	10 (40%)
Some college	7 (28%)
Employment status^a^	
Unemployed	11 (44%)
Part-time	8 (32%)
Full-time	5 (20%)
Opioid used ever	12 (48%)
Age of first substances use (*n*, SD)	15.2 (2.9)
Number of times in jail/prison (*n*, SD)	3.9 (3.6)
Living with HIV	11 (44%)
Housing instability in the last 60 days^a^	6 (24%)
Substances used ever	
Cocaine	17 (68%)
Methamphetamine	8 (32%)
Club drugs^b^	18 (72%)
Hallucinogens^c^	4 (16%)
Cannabis	25 (100%)
Alcohol	18 (72%)
Pain pills^d^	9 (36%)
Heroin	1 (4%)
Cough syrup and codeine	4 (16%)

^a^Missing data

^b^e.g., Ecstasy (MDMA), gamma hydroxybutyrate (GHB), ketamine (‘Special K’)

^c^e.g., Psilocybin (‘magic mushrooms’), lysergic acid diethylamide (LSD/‘acid’)

^d^e.g., OxyContin, Vicodin, Valium, Xanax

### Social network

Older family members and their experiences with substance abuse were frequently cited as a negative influence on how participants perceived “harder” substances and their desire to avoid them. One participant reflected: “*I learned through my own experiences, but it was mostly by was watching others... Like my grandmother, some of her best friends was crackheads. My uncle––I watched him and how drugs messed him up*” **(Houston (HOU), 28)**. In contrast, peers and peer-aged family members (e.g., brothers, cousins) were often identified as influencing participants to initiate consumption of cannabis as well as “hard” drugs; one participant stated, “*Yeah, my sister actually got me doing drugs basically – weed*” **(Chicago (CHI), 22)**. Additionally, peers and peer-aged family members provided participants easy access to cannabis, whether that be through their connections, selling, or using cannabis. “*I have brothers that sell weed and stuff like that, and so, it was easy to maneuver to get that*” **(CHI, 29)**.

Participants described how the social aspects of using cannabis within their networks differ from use of other substances. One participant shared this dynamic, “*I mean, my friends do drugs, but it's not – we're all like the same. It's not like we sit in a big circle and pop ecstasy pills or do lines or do barbs or nothing like that. We may smoke the blunt together, yeah we do that*” **(HOU, 27)**. While cannabis was normalized among friends, mutual or individual usage of “harder” drugs was often perceived negatively; “*Meth is more with other drug addicts and junkies... These people don't care about you. They're drug addicts. We’re all drug addicts. And some of these people don't – most of them don't have a life. Nothing going on for themselves and people have told me they’d rather see me high than sober.”* **(HOU, 24)**. Because of participants’ prior experiences being harmed by people who used “harder” drugs, they developed a heuristic to distrust those peers. These experiences also produced stigmatizing beliefs about the drivers and consequences of “harder” drugs versus cannabis.

While references to the sexual orientations of friend groups were rarely made, when they were, hard drugs use was linked to being LGBT. “*And the methamphetamine is awesome too. And the methamphetamine is used for gay community. It’s like a gay drug. It's like a party drug and it’s for sex*” **(HOU, 24)**. Whereas, with one exception, “*but straight people they just smoke, you know, hang with them and smoke and I snapped out of it like that*” **(CHI, 23)**, cannabis was not linked to sexual orientation at all.

### Need fulfillment

Participant consumption of cannabis was often motivated by a desire to fulfil basic needs (e.g., inducing hunger or sleep) and cope with emotional stressors (e.g., depression). For example, participants consumed cannabis in order to complete productive tasks (e.g., schoolwork and house cleaning). Another participant recalled how it helped them cope with depressive symptoms: “*I wasn't eating properly, I wasn't being nourished properly. So, I started using marijuana*” **(HOU, 26)**. Participants also used cannabis to deal with stress and to achieve a sense of general euphoria or wellbeing. One participant expressed, “*Once I got out of jail I just really had nobody and so it was like I was really, really dependent on weed. Like, that was my life – stress reliever, my coping mechanism, like everything actually*” **(CHI, 25)**.

Finally, participants frequently described using cannabis to cope with a variety of traumas that can be particularly common among populations of young Black MSM with histories of criminal legal involvement. These included recent incarceration, deaths of family members due to violence and other causes, an HIV diagnosis, and assault. “*Later my grandmother got sick and that took another turn – I guess her getting sick and the news about my HIV made me start really using. So, I guess all those like the mix of things was like going on*” **(HOU, 27)**. Cannabis helped them to regulate their emotions and provided relief from negative feelings (e.g., anxiety and depression) in the face of these events. “*You know, if I start getting into my thoughts and my feelings, I, you know, I want to smoke weed just to kind of relax me and calm me down, calm my anxiety down*” **(HOU, 29)**.

Because many participants used cannabis to fulfil needs, including dealing with psychological and physiological conditions (e.g., depression, ADHD, anorexia), they generally framed cannabis not as a “hard” drug but rather as a therapeutic agent or an “herbal” remedy, even replacing prescribed medications. One participant reported that it might be safer that prescribed medications, “*Marijuana is, is edible and it's therapeutic; it’s normal stuff... more like a social therapeutic. Instead of taking half of these psychotropic medications, I’ve found that marijuana does the same thing and it's herbal*” **(HOU, 24)**.

### Knowledge of risk

Participants’ perceived risk of cannabis use versus other, “harder”, substance use was driven by their attitudes toward addiction and their understanding of the potential harmful physiological effects, such as overdose, associated with using non-cannabis substances.

*I just kind of feel like marijuana is the only drug that probably wouldn't kill me. That is why it is the one I use most often. I can use it more and feel safer using it. Cocaine is something I do, but not as often as I do marijuana. I have more fear that I might overdo it with something like cocaine . . . If I think it is milder, I will use it. I have heard some concerning things about what cocaine can do, like effect blood pressure and the heart*. **(HOU, 29)**

Despite participants general understanding of cannabis as being “safer” than other intoxicating substances, their attitudes toward their individual cannabis usage were mixed. Some participants expressed that their cannabis usage was wholly under their control, some described feeling dependent on cannabis consumption, and some matter-of-factly described it as being a non-problematic yet an essential part of their everyday lives. One participant described intentionally pursuing a non-problematic usage regimen: “*Keeping it [cannabis use] under control is my main concern. There's a thin line between checking in with everything and then abusing everything. So just making sure that I keep myself on the positive side of that*” **(CHI, 25)**. Concerns about substance use disorder were pervasive in the sample, with some participants moving from using hard drugs to cannabis because they experienced or observed negative effects of those other drugs that the assumed they could avoid with cannabis. Cannabis thus acts as an effective harm reduction tool for drug users who desire to avoid or minimize use of “harder” substances: “*I don’t like the pills, slouchy and stuff like that. Yeah, it made me real sleepy like, pills like, I was like, I looked like a heroin addict. You know what I mean? Like nodding and stuff, I mean I was like, I don’t like that... After I overdosed no, I only use marijuana*” **(CHI, 24)**. Participants assessed the point at which cannabis usage is therapeutic versus when it becomes problematic according to dynamic personal criteria and experiences with other substances.

Cannabis usage is informed by participants’ social and physical environments, and these networks and needs also influence participants’ experiences with cessation and relapse and/or persistent recovery from substance use including cannabis usage. Another individual demonstrates that their established networks persist despite their decision to cease usage, “*Everything good. We can still hang out. They don’t put any pressure on me to use [cannabis]. They are respecting my decision to not use*” **(HOU, 27)**. Although actual cannabis usage was associated with their peer networks, participants’ knowledge of risk often related back to their own experience of substance usage with cannabis and other drugs or observations of family members.

## Discussion

This paper expands on how social networks and socio-environments can influence drug use; importantly, we emphasize how a majority of participants reported that their peers and family both positively influenced their desire to consume cannabis as well as negatively influenced their desire to consume “heavier” drugs (i.e., stimulants and opioids). Participant narratives of their cannabis usage further emphasized their drug-use behavior as being agentic; rather than passively absorbing influential factors, interviewees endorsed actively considering social contexts and potential consequences when engaging in cannabis-related decision-making and risk-assessment. While most participants endorsed a generally positive relationship with cannabis and did not find their usage disruptive to their daily lives, a small number felt their reliance on cannabis to manage physical and emotional needs was problematic. Social networks as an influencing force for cannabis use is of interest as these same networks could also be leveraged as an intervention for cannabis use disorder [43]. For example, there are several network interventions [44] that could be utilized to promote treatments or health care seeking for cannabis use disorder. A community opinion leader has been successfully utilized for other HIV prevention interventions [45], and is currently being tested to promote COVID-19 prevention behaviors [46].

Criteria for diagnosing cannabis use disorder vary and effective and durable cannabis use disorder treatments are elusive and impact the likelihood that individuals’ will seek treatment or stop using [47], particularly when these individuals are embedded in environments where use is pervasive and accepted. Future research and practice examining the role of mediating factors for cannabis usage, such as anxiety and depression management, has the potential to improve treatments. Our findings add to a growing body of research elucidating how social relationships, including peer and family relationships, as well as negative life events (e.g., incarceration) influence cannabis usage patterns [48]. Shifting statutes around the legality of cannabis and efforts to reduce recidivism highlight the importance of research on the interplay of incarceration, substance use and cessation, and recidivism.

Our findings did not reveal significant differences in how the legality and illegality of cannabis in Chicago and Houston respectively influenced the cannabis use contexts and perceptions of our participants; this may be because all already had experienced incarceration and used substances as a study entry criterion. Analysis of how cannabis consumption behavior differs between legislative jurisdictions should be pursued in future studies, as the landscape of cannabis acceptability and availability is rapidly yet inconsistently evolving across cities.

### Limitations

The interview guide was created to highlight opioid perceptions and consumption in the life course of BMSM who had been incarnated. As such, cannabis perceptions and experiences were not explored in-depth during interviews; however, our analysis is strengthened by not having used leading questions during participant interviews. Additionally, all participants resided in two large metropolitan areas and had a history of incarceration, which may affect generalizability to broader BMSM populations. Last, we did not have other information to corroborate whether participants’ cannabis consumption was problematic, neutral, or effective as self-treatment.

## Conclusion

Our findings reaffirm current understanding of substance use as being influenced by social and familial networks, the desire to satisfy physical and emotional needs, and preconceptions of the risks and benefits involved in “heavy” drug and cannabis usage. Furthermore, we expand on previous findings with the novel observation that participants behaviors related to “heavy” drug and cannabis usage were the result of their own risk assessments versus passive reactions to impersonal socio-environmental factors. Given the expanded availability of cannabis, with expanding legalized recreational use, and marketing that is often directly speaks to the rationales expressed by our participants, further qualitative research will facilitate understanding of the nuanced of risk-assessments people make related to cannabis use. This is particularly important for individuals who were previously incarcerated for cannabis-related charges. Study findings strengthen and expand our understanding of drug consumption-related decision-making, revealing opportunities for future targeted behavioral interventions.

## Data Availability

Data are not publicly available due to protect participant privacy.

## Appendix 1

See Table

**Table 2:** Semi-structured interview guide

Section	Question
Family history	1. Please tell me about your life growing up 2. How would you describe your current relationship with your parents/siblings? 3. How has your family responded, if at all, to your same sex contact?
Current living situation	1. Tell me about your current living situation 2. How do you get by? 3. If there are any, please tell me about other significant events in your life
Personal drug usage	1. Describe your first drug use 2. Describe your first use opioids 3. What was going on in your life at that time? 4. How did the way you use or used opioids (substance and route of administration) change over time? 5. How were you first introduced to injecting? 6. If you used opioids, but avoided injecting, why? How did you make that decision and how did you stick to it? 7. Tell me about times you were tempted to start injecting. What stopped you? 8. Tell me about your experiences with drug treatment 9. What would your ideal drug treatment program look like? 10. Please tell more about your preference for some drugs over others 11. How have you drug using circles changed over time? 12. For those who have never used opioids: (a.) Tell me about your familiarity with opioids. What has kept you from trying them? If you are avoiding them on purpose, why? (b.) What personal experiences have you had with opioid users in your life? (c.) How important is it to you not to use opioids? (d.) What does it take to avoid using opioids?
Withdrawal	1. Describe any withdrawal symptoms that you have related to the drugs you use. Tell me how you managed pain related to withdrawals 2. If you decided to quit the drug, was your ability to quit the drug limited by your withdrawals? Walk me through your thought process 3. How were your schedule and day-to-day activities affected by withdrawal symptoms? 4. Can you count on your social network contacts to help you with withdrawals?
Experiences in jail/prison	1. Have you ever used alcohol or other drugs while in jail? 2. How has incarcerated impacted your drug use? 3. Do periods of incarceration lead you to use more or less drugs once you are out? Why? 4. Has your drug using network changed due to incarceration? 5. What aspects, if any, of being incarcerated help you control your drug use? 6. In general, how does being incarcerated impact you? Emotionally? Housing? Employment? HIV risk? 7. How does being in MSM segregation housing impact your experience of incarceration? 8. How does being in general population impact your experience of incarceration?
Relapse	1. Had you ever quit injecting drugs for a period of time? How long was that period of time? 2. How long did it take you to relapse? 3. In what setting did you relapse? 4. Describe what you were thinking and feeling at the time before you relapsed, and immediately after the relapse

2.

## Abbreviations

BMSM: Black men who have sex with men
HIV: Human immunodeficiency virus
HOU: Houston, Texas
CHI: Chicago, Illinois
DSM-5: Diagnostic and Statistical Manual of Mental Disorders fifth edition
